# Prompt gravity signal induced by the 2011 Tohoku-Oki earthquake

**DOI:** 10.1038/ncomms13349

**Published:** 2016-11-22

**Authors:** Jean-Paul Montagner, Kévin Juhel, Matteo Barsuglia, Jean Paul Ampuero, Eric Chassande-Mottin, Jan Harms, Bernard Whiting, Pascal Bernard, Eric Clévédé, Philippe Lognonné

**Affiliations:** 1 Laboratoire de Sismologie, Institut de Physique du Globe, UMR/CNRS 7154, 1 rue Jussieu, 75238 Paris, France; 2 APC, AstroParticule et Cosmologie, Université Paris Diderot, CNRS/IN2P3, CEA/Irfu, Observatoire de Paris, Sorbonne Paris Cité, 10 Rue Alice Domon et Léonie Duquet, F-75205 Paris Cedex 13, France; 3 Seismological Laboratory, California Institute of Technology, 1200 E. California Blvd., Pasadena, California 91125, USA; 4 National Institute for Nuclear Physics, Sezione Firenze, Via G Sansone 1, Sesto Fiorentino, 50019 and Università degli Studi di Urbino “Carlo Bo“, I-61029 Urbino, Italy; 5 Department of Physics, 2001 Museum Road, University of Florida, Gainesville, Florida 32611-8440, USA

## Abstract

Transient gravity changes are expected to occur at all distances during an earthquake rupture, even before the arrival of seismic waves. Here we report on the search of such a prompt gravity signal in data recorded by a superconducting gravimeter and broadband seismometers during the 2011 Mw 9.0 Tohoku-Oki earthquake. During the earthquake rupture, a signal exceeding the background noise is observed with a statistical significance higher than 99% and an amplitude of a fraction of μGal, consistent in sign and order of magnitude with theoretical predictions from a first-order model. While prompt gravity signal detection with state-of-the-art gravimeters and seismometers is challenged by background seismic noise, its robust detection with gravity gradiometers under development could open new directions in earthquake seismology, and overcome fundamental limitations of current earthquake early-warning systems imposed by the propagation speed of seismic waves.

## Abstract (web summary)

Earthquakes have been theorised to produce gravity signals that may arrive before seismic waves, but until now they had not been detected. Montagner *et al*. have detected prompt gravity signals from the 2011 Tohoku-Oki earthquake thus allowing an early warning of earthquakes before seismic wave arrival.

Earthquakes induce mass redistribution that generates observable changes of the Earth's gravitational field^1^. Static gravity changes have been previously observed by superconducting gravimeters and satellite gravity gradiometers^2^,^3^,^4^,^5^ long after the end of the rupture. These changes are consistent with theoretical predictions^2^,^6^,^7^. In addition to the static signal, a transient gravity change is expected to appear everywhere during the rupture, before the arrival of seismic waves^8^. Such a prompt gravity signal is in general expected to be very small compared with the background seismic noise that affects all gravity sensors (see section 4.1 of ref. 8). Thus, to maximize the chance of detection, it is necessary to consider a very large earthquake recorded in a low-noise environment at distances large enough to capitalize on the time between rupture onset and arrival of seismic waves, but short enough to not be challenged by the rapid distance decay of the gravity field.

Here we report on the search for a prompt gravity signal during the rupture of the 2011 Mw 9.0 Tohoku-Oki earthquake in data recorded by a superconducting gravimeter in the underground Kamioka Observatory and five nearby broadband seismometers from the Japanese network F-net. Through a statistical blind analysis, we find significant evidence of the presence of a prompt gravity signal in the data set. This finding is further supported by the order-of-magnitude agreement between the signal amplitudes observed and those predicted by current theory. The measurement of gravity precursors to earthquake shaking with next-generation sensors could contribute to earthquake early warning and provide new insights on rupture initiation.

## Results

### Data

**Figure 1:**
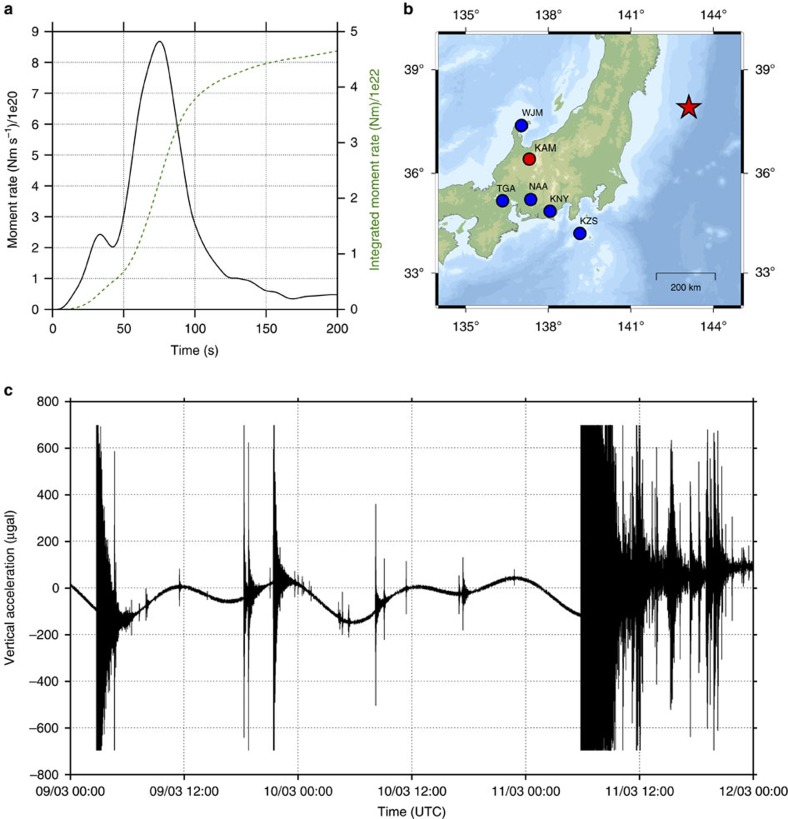
The 2011 Tohoku-Oki earthquake and gravity changes recorded by the Kamioka superconducting gravimeter. (**a**) Seismic moment (dashed line) and moment rate (solid line) time-functions of the Tohoku-Oki earthquake^12^. (**b**) Locations of the epicentre (red star), the Kamioka Observatory (red circle) and its five nearest F-net seismic stations (blue circles). (**c**) Three days of gravimetric recording at Kamioka Observatory, starting on 9 March 2011.

The 11 March 2011 Tohoku-Oki earthquake occurred off the Pacific coast of northern Honshu, Japan^9^,^10^. The rupture onset time is *t*_eq_=05:46:21 UTC and the epicentre coordinates are 38.19° N, 142.68° E (ref. 11). The moment rate function^12^ has an overall duration of more than 2 min and reaches its maximum over about 1 min (Fig. 1a). Based on the theory developed by Harms, J. *et al*.^8^ (see also the ‘Discussion' section), we expect a prompt gravity signal amplitude of a fraction of μGal at ∼65 s after the rupture onset, just before the arrival of the seismic P-waves at the instruments.

The superconducting gravimeter is located in the underground Kamioka Observatory and is operated by the National Astronomical Observatory of Japan (Mizusawa VERA Observatory) in the framework of the Global Geodynamics Project^13^. It is the only superconducting gravimeter in Japan that records at sufficiently high sampling rate for our purposes (1 Hz). The distance and azimuth between the Tohoku-Oki earthquake epicentre and the Kamioka station are ∼510 km and 250°, respectively (Fig. 1b). The P-wave arrival time at the Kamioka station identified on the gravimetric record is *t*_P_≃05:47:35 UTC. The data, recovered from the GGP1 channel, contains an analogue anti-aliasing filter (eighth-order Bessel filter, with corner frequency of 61 mHz).

We also consider the five F-net stations closest to Kamioka Observatory to validate the Kamioka station gravity observations. Their locations are shown in Fig. 1b. They are either STS-1 or STS-2 broadband velocimeters, and the acceleration waveforms are obtained through single derivation. The distance from the Tohoku-Oki epicentre and the travel times of P-waves exceed, respectively, 500 km and 65 s for all stations.

### Statistical analysis of the Kamioka gravimeter data

The goal of our analysis is to assess the existence of a transient gravity signal within the ∼65 s-long interval between the rupture onset time *t*_eq_ and the P-wave arrival time *t*_P_. We first focus our analysis on the superconducting gravimeter recording. A preliminary analysis (see Supplementary Fig. 1) suggested the presence of a transient gravity signal whose amplitude does not obviously stand above the background microseism noise.

To achieve an objective signal detection assessment in such a low signal-to-noise environment, avoiding any human bias, we have developed a completely independent statistical detection procedure whose parameters are tuned using only the background data that excludes data recorded in the period around the Tohoku-Oki earthquake (60 days of background data, from 1 March 2011 05:46:00 UTC to 11 March 05:46:00 UTC and from 12 March to 30 April). Only once the optimal analysis parameters are determined, we ‘open the box' and apply the detection procedure to the data around the Tohoku-Oki rupture onset time (hereafter denoted as the event interval). We then compute its statistical significance, without any posterior re-tuning of the analysis parameters. This kind of analysis is called blind since, during the tuning stage, the results (strength and statistical significance of the signal at the time of the earthquake) are not known. The technique is described in detail in the following sections and in the Supplementary Information.

Our analysis of the gravimetric data does not rely on a specific model of the transient signal, only on the theoretical expectation that it grows monotonically^8^,^14^. The slow trend in the gravimeter data *x*(*t*), which includes tidal perturbations, is fitted by polynomial functions *f*_*d*,*T*_(*t*) of degree *d* over time segments [*t*_start_, *t*_end_] of duration *T*. For the event interval, we set *t*_end_=*t*_eq_. Once the fitting coefficients are computed, we extrapolate the fitted function beyond *t*_end_. We then compute a reduced gravity signal 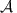 by averaging the residuals between data and extrapolating function over a time window [*t*_1_, *t*_2_], with *t*_1_≥*t*_end_ and *t*_2_≤*t*_P_, by a discretized version of:


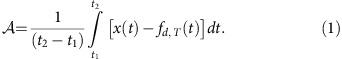


We stress that 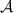 is not the value of the gravity signal at a specific time, but an overall measure of the signal strength, whose statistical significance will be assessed. We define 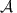 by a simple average because the transient gravity signal is expected to grow monotonically, as the second time integral of the seismic moment function^8^,^14^. The microseismic noise present in the residuals with periods between 5 and 10 s is partially filtered out by the average in equation (1) by setting *t*_2_−*t*_1_=30 s. Since the transient gravity signal is expected to increase rapidly with time^8^, the strongest signals should be contained in the last part of the time window [*t*_eq_, *t*_P_]. For this reason, we set *t*_2_=*t*_end_+65 s. For the event interval, since *t*_2_=*t*_P_−9 s, the analysis is not corrupted by P-waves.

The analysis has two free parameters, both related to fitting, namely the duration of the trend-fitting segment, *T*, and the polynomial order, *d*. We tune their value by a blind procedure applied to data from the background. To maximize the number of background-reduced signals, we overlap the segments and repeat the fitting procedure every 10 s on the background data. To assess the statistical significance of the prompt gravity signal during the event interval, we need to discard the background data contaminated by other earthquakes. We retain only the segments with noise characteristics similar to the event interval. More precisely, defining *σ*_Tohoku_ as the standard deviation of a 30 min-long time window preceding the Tohoku event, we keep segments if the standard deviation of their trend-fitting section *σ* fulfills 0.75*σ*_Tohoku_<*σ*<1.25*σ*_Tohoku_ and if the standard deviation of their extrapolation section *σ*_extr_ obeys *σ*_extr_<10*σ*_Tohoku_. These choices, still performed in a blind way (using only the background data and not the Tohoku signal), realize a reasonable trade-off between the available amount of background data and its statistical consistency with the Tohoku-Oki data.

**Figure 2:**
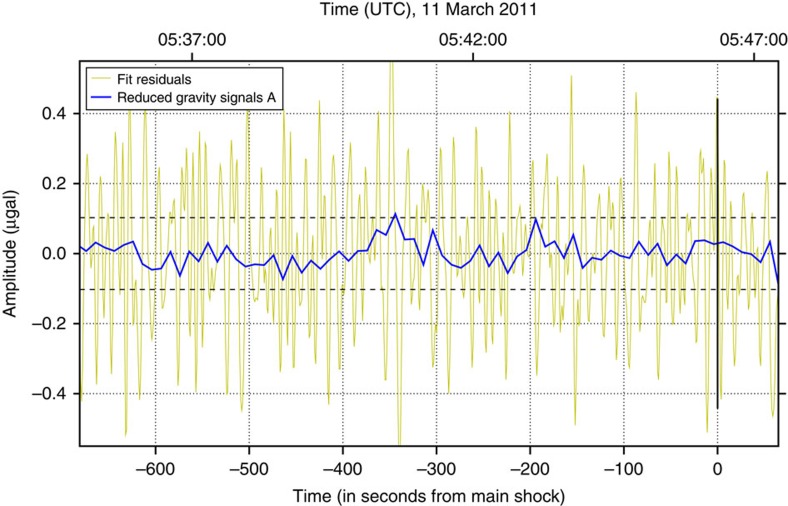
Time series of residuals and reduced gravity signal. Yellow line: residuals obtained from least squares polynomial fit (*d*=2, *T*=690 s). Blue line: reduced gravity signal 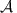 around the occurence of the Tohoku-Oki earthquake (vertical solid line). The horizontal dashed lines indicate 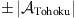. The 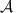 value exceeding the upper dashed line near −350 s is considered an outlier.

**Figure 3:**
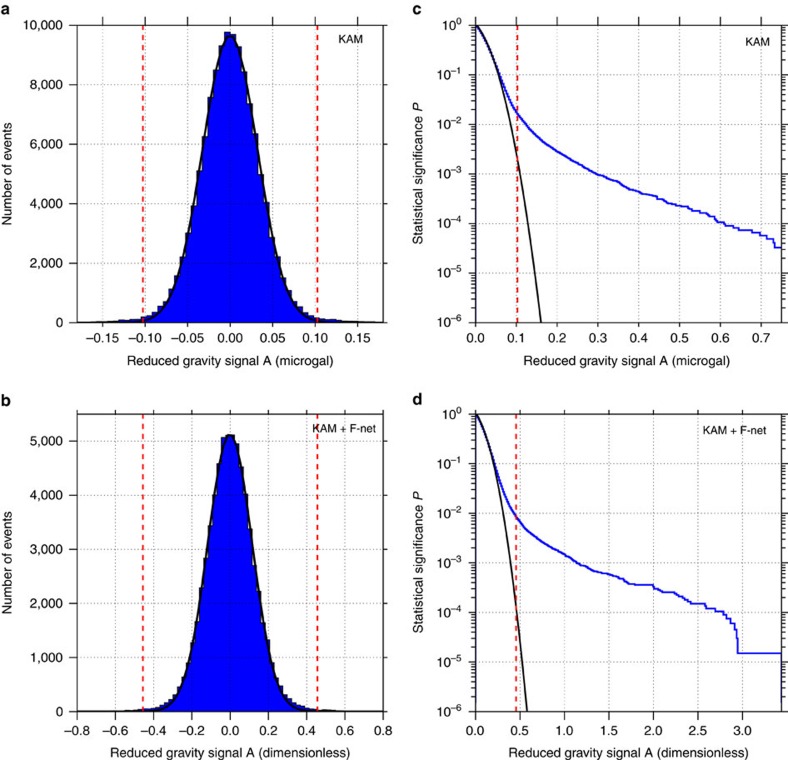
Statistical analysis of gravity and seismic data. Distribution of the background reduced gravity signals 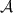 for (**a**) the superconducting gravimeter record only (*d*=2, *T*=690 s) and (**b**) the weighted stack of gravimeter and broadband seismometers records (*d*=1, *T*=1,900 s). The dashed red vertical lines represent the reduced gravity signal for the event 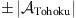. Note that the weighted stack is dimensionless. Empirical cumulative probability function (probability for a signal to exceed 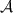) (blue curve) for (**c**) the gravimeter record only and for (**d**) the weighted stack record. The probability to obtain a signal larger than 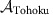 (dashed vertical line) is 1.6% for (**c**) and 0.8% for (**d**). In all plots, the black curve corresponds to the best fitting Gaussian distribution.

We computed the reduced gravity signal 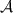 of all selected background time intervals for a range of values of parameters *d* and *T*. The parameter values providing the best fit, that is, the smallest variance of 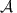, are *d*=2 and *T*=690 s. The reduced gravity signal time series for the Tohoku-Oki data with this optimal parameter setting are shown in Fig. 2. The resulting gravity signal strength is 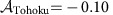 μGal. It is compared with the background distribution in Fig. 3a. The cumulative probability, that is, the probability for a signal strength to exceed 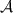, is displayed in Fig. 3c. We note that the tails of the background distribution are not Gaussian. The fraction of background intervals with a larger 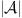 gives the statistical significance *P* of the result. The reduced gravity signal equals or exceeds 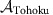 for 2,061 out of 127,885 background intervals, hence the probability that the signal strength in the Tohoku interval arises from the background fluctuations is *P*=2,061/127,885=1.6% (dashed vertical line in Fig. 3c). In other words, we can reject the null hypothesis that the observed Kamioka signal is due simply to background fluctuations with 98.4% confidence.

### Statistical analysis including broadband seismic data

To gather further evidence of a transient gravity signal, we extend the analysis to five broadband F-net stations with similar epicentral distances and excitation amplitude (far from radiation nodes). The preliminary analysis of the four closest stations is shown in Supplementary Fig. 2. Here we describe the independent blind analysis. The seismic waveforms are filtered with the same anti-alias filter as the Kamioka gravimetric data. In addition, both gravimetric and seismic data are filtered with a causal high-pass fourth-order Bessel filter with corner frequency 1 mHz. Even though the F-net stations are noisier than the Kamioka gravimeter at long periods, one way to reduce the propagating noise on the network and to enhance the detection of a transient gravity signal, recorded in theory at the same time by all stations, is to stack the recordings. To achieve an optimal stack despite variable signal-to-noise levels at each station, the stack is weighted by the expected (theoretical) signal amplitude (see next section) and the variance of noise at each station, as follows:


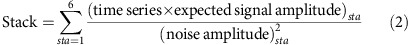


The blind statistical detection procedure is then repeated on the stacked data. New background data are drawn by applying the same selection criteria as before to the stacked data. The parameter values providing the smallest variance of 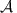 in the background data are in this case *d*=1 and *T*=1,900 s. We compare the background distribution of 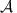 to the value of 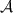 in the Tohoku interval in Fig. 3b. The fraction of background intervals with a larger 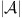 than the Tohoku interval gives the statistical significance *P*=548/66,804=0.82% (dashed line in the cumulative probability display in Fig. 3d). Thus, we can reject the null hypothesis with more than 99% confidence.

An alternative analysis, a matched filter detection using as template the theoretical solution presented in the next section and the whole 65 s-long interval before the P-waves arrival, gives comparable results.

## Discussion

**Figure 4:**
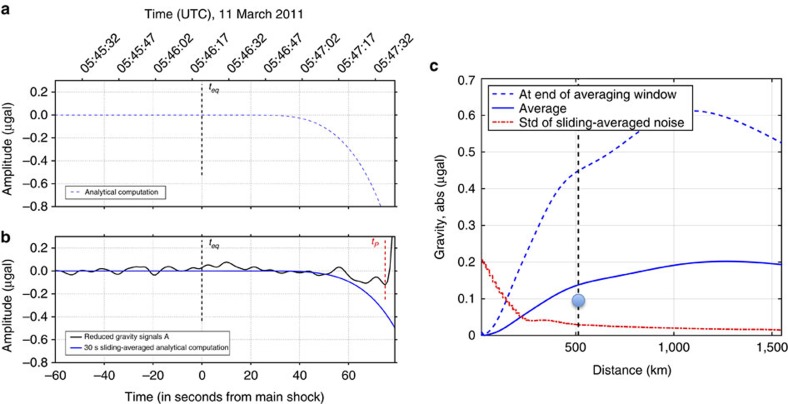
Comparison between observations and a theoretical model. (**a**) Analytical computation of the gravity perturbation produced at Kamioka by the Tohoku-Oki earthquake. (**b**) The 30 s sliding-averaged theoretical prediction (solid blue) and observed reduced gravity signals (black). (**c**) Gravity perturbation near the P-wave arrival, with (solid blue) and without (dashed blue) sliding-average, as a function of distance to the earthquake centroid. The duration of the averaging window is increased with increasing distance to the source. The red line is the standard deviation of the sliding-averaged background noise. The vertical dashed line marks the distance of the Kamioka Observatory, the dot indicates the observed value of the reduced gravity signal at Kamioka.

Past models of gravity perturbations generated by earthquakes have been mostly restricted to quasi-static gravity changes^15^. A first-order analytical model of dynamic gravity transients from earthquakes^8^ is available for a point-shear dislocation in infinite, homogeneous, non-self-gravitating elastic media. Numerical simulations^8^ indicate that the model stays adequately accurate even after seismic waves have reached the Earth's surface^14^. This analytical model is used to predict the gravity perturbation from the Tohoku-Oki earthquake at the Kamioka Observatory and at the location of the F-net stations. More complete models^14^ will be implemented in further studies. Figure 4a shows the analytical perturbation at the Kamioka Observatory, filtered with the anti-alias filter described in the previous section. We then apply the 30 s sliding average to the filtered analytical prediction (Fig. 4b), and obtain 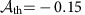 μGal at the Kamioka Observatory. This value is within 50% of the measured value, 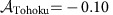 μGal, and has the same sign. We therefore conclude that the observation presented here is consistent with the first-order simple analytical model.

Figure 4c shows the analytical prediction of the gravity perturbation just before the P-wave arrival as a function of distance to the earthquake centroid, evaluated on the great circle connecting the earthquake centroid and Kamioka. While prompt gravity perturbations decrease with increasing distance as *r*^−4^ (ref. 8), the gravity perturbation at P-wave arrival increases for distances smaller than ∼1,000 km since the source-time function rises quickly in the beginning of the event (see Fig. 1a). At longer distances, the perturbation decays due to the source-time function reaching its maximum after about 60 s, and due to the angular dependence of the signal. To roughly estimate a signal-to-noise ratio, we filter by a sliding-average the predicted signal and the residual of our preferred polynomial fit to the Tohoku-Oki data. The averaging window duration, *t*_2_−*t*_1_, is scaled with distance to the centroid such that, at the distance of the Kamioka Observatory, it is equal to the value *t*_2_−*t*_1_=30 s used in our main analysis of the Kamioka data. At each distance, the noise level is defined as the standard deviation of the sliding-averaged residuals before *t*_start_, and the reduced gravity signal value is computed on the distance-scaled window [*t*_1_, *t*_2_]. Both quantities are shown in Fig. 4c by red and blue curves, respectively. Up to a distance of about 250 km, a strong reduction in noise level is observed, mostly due to increasingly efficient filtering of the oceanic microseisms by the increasing sliding-average window duration. At larger distances, the noise level decays less rapidly. This calculation suggests that sensors with noise level similar to the Kamioka gravimeter, at a broad range of distances, may help to detect the transient gravity perturbation. A complete analysis of all F-net seismic stations will be presented elsewhere.

In conclusion, we have reported the search of the prompt gravity signal produced by an earthquake, a gravitational precursor to ground shaking. The search is based on a blind statistical detection algorithm and yields a statistical significance ∼99% when combining the Kamioka gravimeter and five F-net stations. The amplitude of the detected signal agrees within a factor of 1.5 with a theoretical prediction.

Our analysis illustrates that detection of transient gravity signals is hindered by the background seismic noise. Therefore, to improve the capability to measure a prompt gravity signal, a new class of instruments is necessary. Very sensitive gravity strainmeters, able to measure the gravity gradient between seismically isolated test masses, are currently under study^8^,^16^. The sensitivity of these new instruments remains to be demonstrated and an optimal signal detection strategy remains to be designed. Nevertheless, the robust detection of transient gravity signals by a network of such instruments has the potential to reduce the time required to declare a warning, thus opening new directions in earthquake seismology, enabling faster earthquake magnitude estimation (which currently takes up to several minutes^17^,^18^,^19^) and complementing earthquake early-warning systems currently based on seismic and geodetic networks^20^,^21^,^22^,^23^.

## Methods

### Blind statistical analysis

Two steps of the blind statistical detection procedure are illustrated for the Kamioka records in Supplementary Figs 4–6. The blind analysis in the main text for Kamioka and F-net stations at similar epicentral distances follows the same procedure.

### Data availability

The data from the superconducting gravimeter of Kamioka Observatory are publicly accessible from the Global Geodynamics Project web site (http://www.eas.slu.edu/GGP/tohoku2011.html) or upon request to Yoshiaki Tamura. The F-net seismic data are available on the web site: http://www.fnet.bosai.go.jp/waveform/?LANG=en. All codes were written for this study and a copy of the data are available on GitHub at the following URL: https://github.com/kjuhel/prompt-gravity.

## Additional information

**How to cite this article:** Montagner, J.-P. *et al*. Prompt gravity signal induced by the 2011 Tohoku-Oki earthquake. *Nat. Commun.* **7,** 13349 doi: 10.1038/ncomms13349 (2016).

**Publisher's note**: Springer Nature remains neutral with regard to jurisdictional claims in published maps and institutional affiliations.

## Supplementary Material

Supplementary Information Supplementary Figures 1-6, Supplementary Methods and Supplementary References.
