# Supplementary material for: Prompt gravity signal induced by the 2011 Tohoku-Oki earthquake
Source: Nat Commun. 2016 Nov 22;7:13349. doi: 10.1038/ncomms13349 (PMC5121411; doi:10.1038/ncomms13349)
Supplement: Supplementary Information — Supplementary Figures 1-6, Supplementary Methods and Supplementary References. [file ncomms13349-s1.pdf]

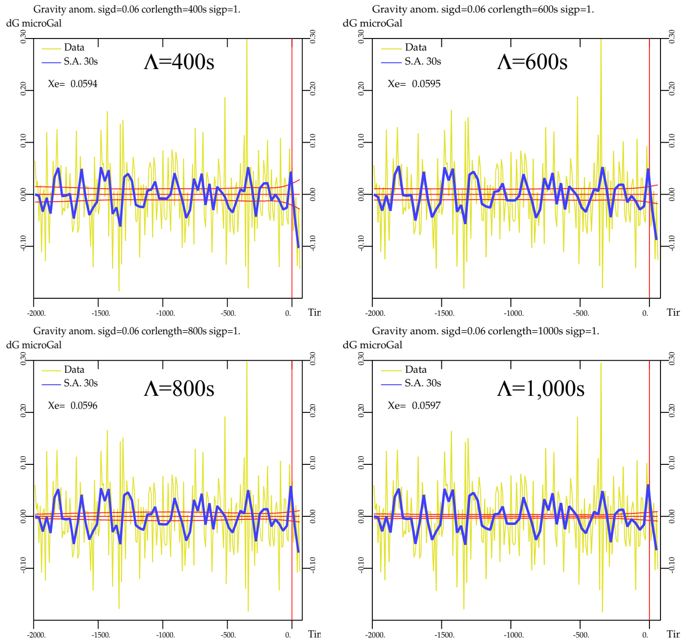

Supplementary Figure 1: Residuals of the Kamioka gravity data (yellow line) before the Tohoku event for different correlation times Λ from 400 s to 1000 s. The vertical red line corresponds to the rupture onset time, t_{eq}. P-waves arrive approximately at $t_P = t_{eq} + 72$ s. The fitting curve is calculated up to t_{eq} and extrapolated analytically between t_{eq} and t_P. The upper and lower red curves indicate the *a posteriori* uncertainties (diagonal terms of the *a posteriori* covariance function of parameters). The horizontal scale is in seconds and the vertical scale in μGal ($10^{-8}\, m/s^2$). Residuals smoothed by sliding average (S.A.) over 30 s are shown as a blue curve.

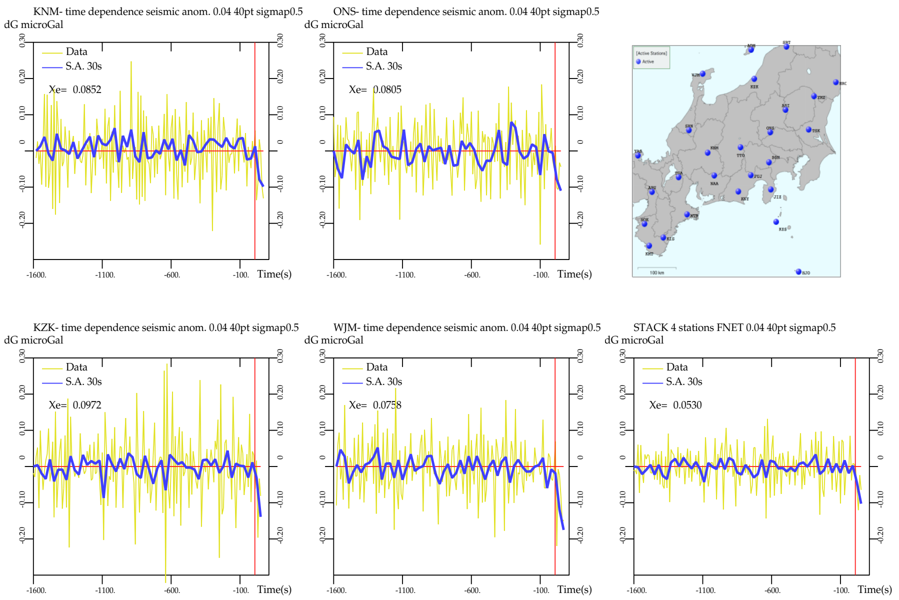

Supplementary Figure 2: Four left figures: yellow curves are residuals of vertical-component broadband seismograms at F-net stations KNM, ONS, KZK and WJM relative to the smoothed curve obtained by the method described above. The horizontal scale is in seconds and the vertical scale in μGal ($10^{-8}\, m/s^2$). Blue lines are residuals filtered by a 30 s sliding average. Bottom right: residuals and smoothed residuals of the stack of the four seismograms. Top right: map of the F-net network.

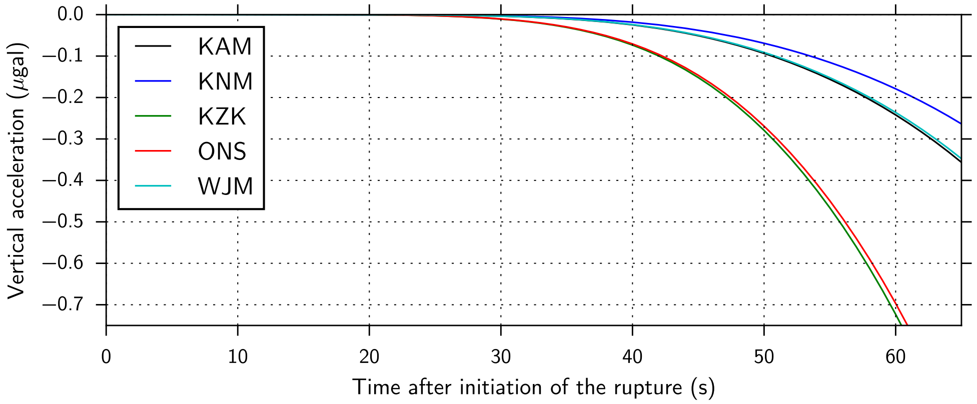

Supplementary Figure 3: Theoretical calculation of the gravity signal at different stations of F-net, KNM, KZK, ONS, WJM, and Kamioka gravimeter station KAM. The anti-alias filter described in the main text is applied, as in Figure 4(a).

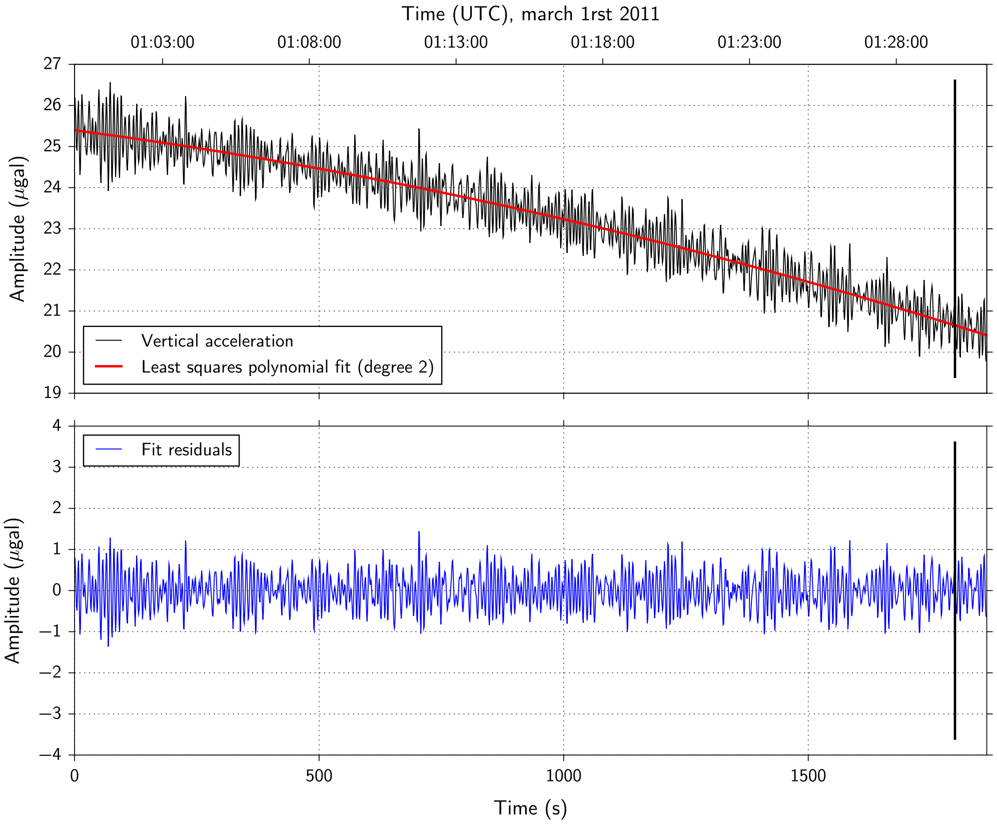

Supplementary Figure 4: Example of signal detection method for a typical time interval. This figure shows that the signal is largely dominated by the microseismic noise, which is reduced by applying a 30 s-sliding average window. Top : Least squares polynomial fit (red) applied on a background time interval signal (black). In this example, the degree of the fitting polynomial is 2, and its coefficients are those that minimize the sum of squared errors in the 1800 seconds-long interval (up to the vertical line). Once the coefficients are determined, we extrapolate the fitting polynomial after the vertical line, for 65 seconds. Bottom : Fit residuals, i.e. the difference between the fitting polynomial and the vertical acceleration. If its standard deviation differs significantly from the standard deviation of the event interval σ_{Tohoku}, the corresponding time interval is rejected, and the reduced gravity signal $\mathcal{A}$ is not computed. Defining σ as the standard deviation of the fit residuals up to the vertical line and σ_{extr} as its standard deviation after the vertical line, the interval is rejected if it fails to satisfy $0.75\,\sigma_{\text{Tohoku}} < \sigma < 1.25\,\sigma_{\text{Tohoku}}$ and $\sigma_{extr} < 10\,\sigma_{\text{Tohoku}}$.

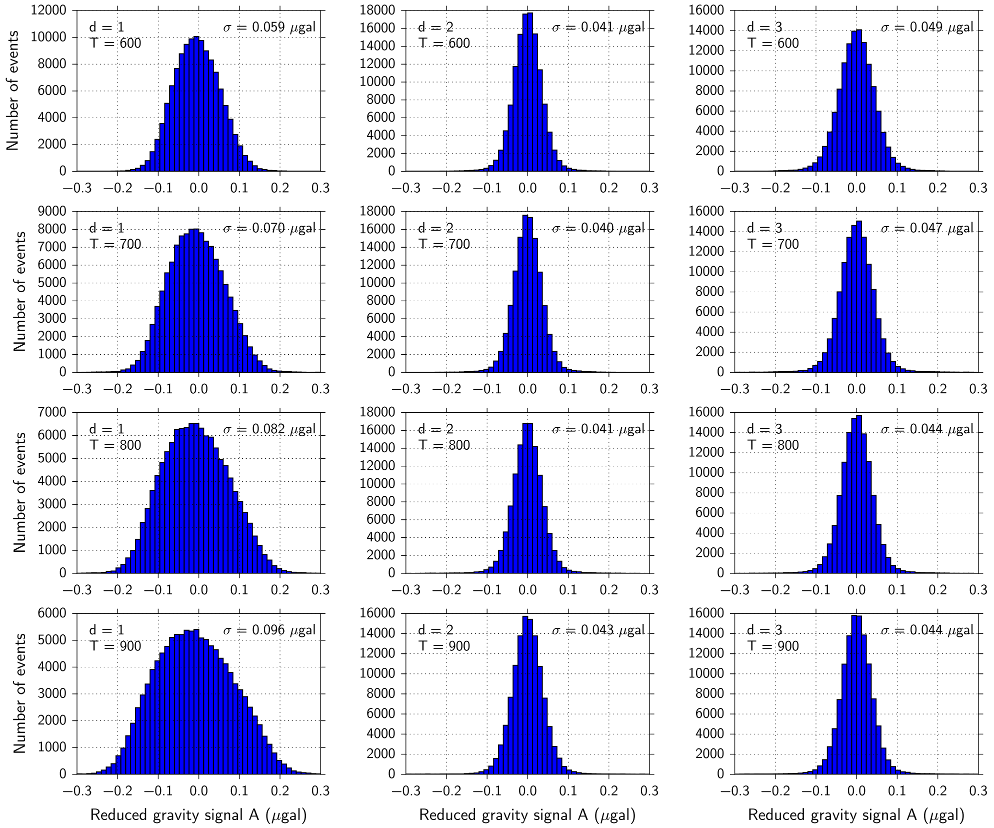

Supplementary Figure 5: Distributions of the reduced gravity signals for the background data, as a function of the time window T and of the polynomial degree d used to fit the acceleration on background time intervals. The computation of the reduced gravity signal $\mathcal{A}$ is performed for all the selected intervals. We explore the whole parameter space (T, d) but only some examples are displayed in this figure. For a given time window T, if d is too small, the fit cannot represent the complexity of the data, whereas if d is too large, its extrapolation diverges quickly. In both cases, the resulting $\mathcal{A}$ distributions are broadened. Thus, for this rough parameter space exploration, the smallest variance of $\mathcal{A}$ is obtained for d=2 and T=700 seconds (not far from the optimal parameter setting, d=2 and T=690s, obtained with a more thorough, refined exploration).

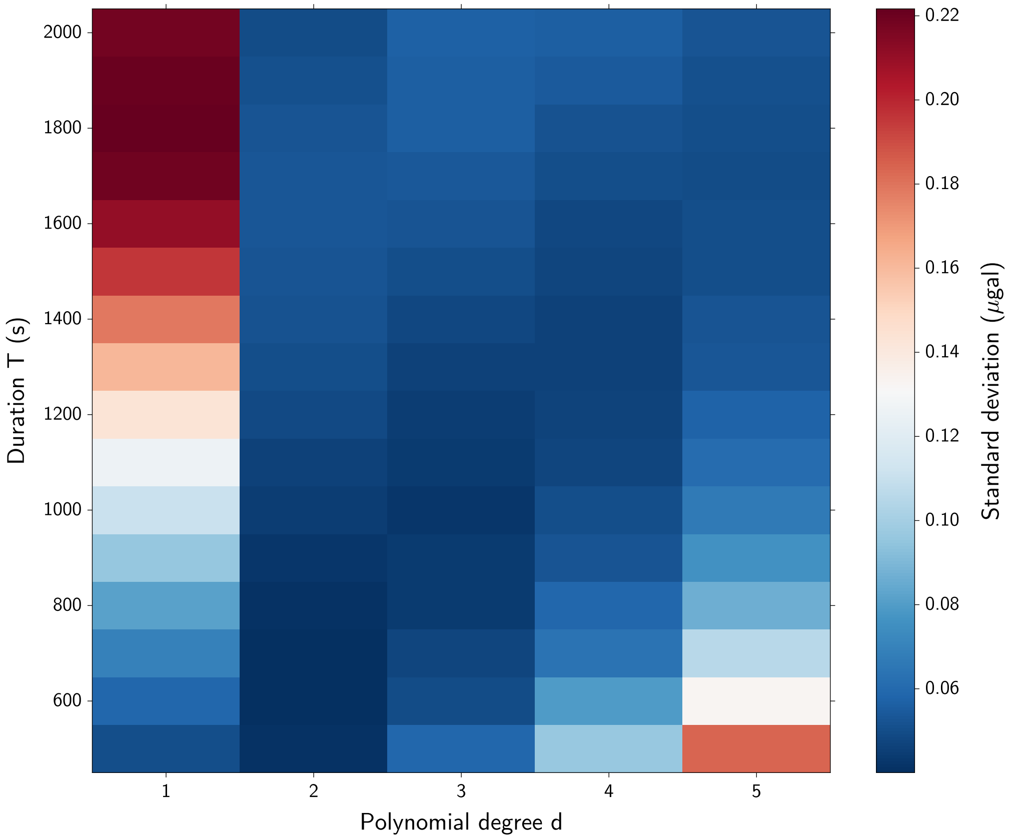

Supplementary Figure 6: Standard deviation of the reduced gravity signals for background data, as a function of the time window duration T and the polynomial degree d used for the fit. The (rough) parameter space exploration extends the one presented in Supplementary Figure 5, the smallest variance of $\mathcal{A}$ is still obtained for d=2 and T=700s.

Supplementary Methods

Preliminary analysis of gravimetric and seismic datasets

A preliminary analysis of the gravimetric and broadband seismic data was performed by fitting the data $x(t)$ by a slowly varying continuous function $f_{synt}(t)$, up to t_{end}, and then extrapolating $f_{synt}(t)$ after t_{end} up to the arrival of P-waves. We adopt the Bayesian fitting approach described by [1], which avoids side effects such as acausal signal and ringing phenomena, and requires the choice of a correlation time Λ of the noise and a fitting interval T. The errors on the fit can be derived from the diagonal terms of the *a posteriori* covariance function of parameters.

- Kamioka dataset

The differences between data and fit are shown in Supplementary Figure 1 for Λ ranging from 400 s to 1000 s and for $T = 2000$ s. The data are decimated to a sampling rate of 0.1 Hz in order to mitigate the microseismic noise and to enhance the low frequency components. The residuals are largely dependent on the choice of Λ. They can be as large as $-0.2\,\mu$Gal for the shortest Λ, and decrease as Λ increases. We smoothed the residuals by applying a sliding average window of 30 s, which further reduces the microseismic noise. The averaged residual (blue line) is in the range -0.15-0.1μGal, slightly larger than other anomalies before t_{end}, but the result is largely dependent upon the choice of the correlation time Λ and the fitting time T.

This preliminary search suggests the presence of a signal, but is not sufficient to assess a statistical significance. Since the number of independent parameters is not defined in the fitting method, in order to be independent of the choice of the fitting parameters, we designed the *blind* statistical analysis described in the main text. The statistical analysis uses polynomial functions to fit the slow trend, for the sake of simplicity and computational efficiency. The blind analysis with polynomials is different and independent from the preliminary analysis.

- Broadband seismic data of the Japanese network F-net

We consider the broadband seismic data of four stations close to Kamioka with the best signal-to-noise ratio. They are processed with the same method

described above, with a correlation time of 400 s. Since we look for an instantaneous gravity effect (no propagation), we stack these data to improve the signal-to-noise ratio (Supplementary Figure 2). The theoretical predictions as in the main text are presented in Supplementary Figure 3.

Blind statistical analysis of the Kamioka dataset

Two steps of the blind statistical detection procedure are illustrated for the Kamioka records in Supplementary Figures 4 to 6. The blind test in the main text, for Kamioka and F-net seismic stations at similar epicentral distances follows the same procedure.

Supplementary References

[1] Tarantola, A. & Valette, B. Generalized nonlinear inverse problems solved using least squares criterion. *Rev. Geophys. Space Phys.* **20**, 219–232 (1982).
